# Prevalence of sleep disturbance and the association between poor disease control in people with ankylosing spondylitis within the Australian clinical setting (ASLEEP study): a real-world observational study using the OPAL dataset

**DOI:** 10.1007/s10067-021-05953-8

**Published:** 2021-11-26

**Authors:** Kathleen Tymms, Belinda E. Butcher, Tracey L. Sletten, Tegan Smith, Catherine O’Sullivan, Geoffrey Littlejohn, Ricky Sadler, Rebecca Tronnberg, Hedley Griffiths, on behalf of the OPAL consortium

**Affiliations:** 1 OPAL Rheumatology Ltd, Sydney, NSW Australia; 2 Canberra Rheumatology, 9/40 Marcus Clarke St, Canberra, ACT 2601 Australia; 3 University of New South Wales, Kensington, NSW Australia; 4 WriteSource Medical Pty Ltd, Lane Cove, NSW Australia; 5 Turner Institute for Brain and Mental Health, School of Psychological Sciences, Monash University, Clayton, VIC Australia; 6 Department of Medicine, Monash University, Clayton, VIC Australia; 7 Novartis Pharmaceuticals Australia Pty Ltd, Macquarie Park, NSW Australia; 8 Barwon Rheumatology Service, Geelong, VIC Australia

**Keywords:** Ankylosing spondylitis, Insomnia, Interleukin 17A inhibitors, Sleep apnoea, Sleep disorders, Tumour necrosis factor inhibitors

## Abstract

**Introduction:**

Sleep disturbance and fatigue are commonly reported in ankylosing spondylitis (AS) but specific prevalence and the relationship to disease control are unknown.

**Method:**

This retrospective non-interventional observational study of data from the OPAL dataset included patients with AS (ICD code M45, M45.0 or M08.1), aged 18 to 95 years and had completed ≥ 1 sleep questionnaire between 1 January 2019 and 30 September 2020. The prevalence of insomnia and obstructive sleep apnoea were assessed using the Insomnia Severity Index (ISI) and Multivariate Apnoea Prediction Index (MAPI), respectively. Propensity score (PS) matching based on sex, age and symptom duration increased comparability between patients administered tumour necrosis factor inhibitors (TNFi) and interleukin 17A inhibitors (IL-17Ai).

**Results:**

Four hundred ninety-five patients were included. The mean ISI total score in the overall population was 8.6 ± 6.2. Self-reported moderate or severe clinical insomnia was present in 16% and 3.2% of patients, respectively. The mean MAPI score was 0.4 ± 0.3, self-reported apnoea was identified in 31.5% of patients and the mean FACIT-Fatigue score was 36.1 ± 10.7. In the PS matched population, the only treatment-related difference was the mean MAPI score (IL-17Ai 0.4 ± 0.3 and TNFi 0.3 ± 0.2, *p* = 0.046). Those with poor disease control (BASDAI ≥ 4) were more likely (odds ratio [OR] 7.29, 95% CI 2.37 to 22.46, *p* = 0.001) to have a greater severity of insomnia symptoms than those with good disease control.

**Conclusion:**

In this real-world AS cohort, poor disease control was associated with sleep disturbance. Little difference in sleep disturbance was observed between biologic TNFi and IL-17Ai treatment.

**Table:** 

**Key Points**
*• Sleep disturbance and fatigue are common in patients with ankylosing spondylitis.* *• In our real-world cohort, self-reported apnoea was reported in one-third of patients; and one in five patients reported moderate to severe insomnia.* *• Those with poor disease control were more likely to experience greater sleep disturbance than those with good disease control.*

## Introduction

Ankylosing spondylitis (AS) is characterized by inflammation of entheseal structures and the adjacent bone marrow resulting in structural damage and new bone formation in spine, sacroiliac joints and other entheseal sites. As the control of inflammatory back pain and stiffness improves with the introduction of biologic disease modifying antirheumatic drugs (bDMARDs), clinicians are now turning their attention to comorbidities and extra-articular manifestations of AS, including sleep disturbances and fatigue. Sleep quality and fatigue are important contributors to overall quality of life [1].

A sleep disorder is a condition that interferes with the quality, timing and duration of sleep. The most common types of sleep disorders include insomnia, sleep-related breathing disorders such as sleep apnoea, circadian rhythm disorders and sleep movement disorders. Others have reported that sleep disorders (including difficulties with sleep quality, sleep latency, sleep efficiency and sleep medication use) are more prevalent in AS patients compared to the general population, particularly in AS patients with high disease activity [1]. Sleep disturbance and fatigue are common features of AS [2, 3] with sleep disturbance prevalence estimates varying between 59 and 90% [1, 3, 4]. Sleep disturbances are associated with increased pain, fatigue, disease activity, mood and depressive symptoms [1, 4].

The causes of sleep disturbance in patients with AS are not fully understood and are likely to be multifactorial. For example, pathological changes caused by AS including spinal deformities may cause pain, and thoracic manifestations affecting the chest wall, airways, lung parenchyma, heart and great vessels with cervical spine deformities can affect upper airways, for example via limited thoracic expansion [5]. Similarly, depression which has a higher prevalence in patients with AS influences sleep disturbance, although there is a bidirectional relationship with depression, so it is difficult to understand which is causal [6]. The pro-inflammatory cytokines (TNFα and IL-1) have been implicated in sleep regulation, enhancing non-rapid eye movement sleep (NREM) and inducing sleepiness [7]. There is also an association between AS and obstructive sleep apnoea, which in turn is linked with excessive daytime sleepiness, hypertension, cardiovascular disease and fatigue [2]. Interestingly, TNFα is elevated in patients with sleep apnoea (in the absence of any concomitant rheumatological diagnosis) [8], and continuous positive airway pressure has been shown to decrease soluble TNF in this setting [9]. There is some evidence to suggest that the frequency of obstructive sleep apnoea is lower in patients receiving tumour necrosis factor inhibitors (TNFi) compared to those not taking TNFi, although this study was small [10].

While there have been some cross-sectional studies conducted elsewhere [5], to date, there has not been studies performed to assess the prevalence of sleep disturbances in a real-world cohort of patients with AS in Australia. To address this gap, the primary objective of this study is to estimate the prevalence of sleep disturbances in patients with AS who are included in the OPAL (**O**ptimizing **P**atient outcomes in **A**ustralian Rheumato**L**ogy) dataset. The secondary objectives included the impact of therapy for AS on sleep outcomes and correlations between disease activity and fatigue with sleep disturbance.

## Method

### Study design

This is a cross-sectional study of observational data embedded within the OPAL dataset. The OPAL dataset contains de-identified information captured during routine clinical care using purpose-built worksheets in the Audit4 electronic medical record (EMR) [11, 12]. A protocol for this study was developed and approved by the institutional ethics committee prior to analysis. The protocol was not registered on the Open Science Framework, nor was it published. A statistical analysis plan was also developed and approved prior to analysis. No post hoc changes to the statistical analysis plan were made.

Data from patients with a physician diagnosis of AS (ICD code M45, M45.0 or M08.1) in the OPAL dataset, were alive at the time of the data cut and between 18 and 95 years and had completed at least one Insomnia Severity Index (ISI) or Multivariate Apnoea Prediction Index (MAPI) questionnaire between 1 January 2019 and 30 September 2020 were included in the study. Variables used in this study included the patient’s demographics (sex, age, body mass index, symptom duration [defined as the date of sleep questionnaire administration minus the date of onset of symptoms], line of biologic therapy), disease severity (Bath Ankylosing Spondylitis Disease Activity Index [BASDAI], erythrocyte sedimentation rate [ESR], c-reactive protein [CRP]), treatment (corticosteroid, NSAID, bDMARD), the presence of co-morbid depression (ICD codes F06.32, F10.8, F11.8, F13.8, F14.8, F15.8, F16.8, F18.8, F19.8, F32, F33 or F34.1) and responses to patient-reported outcome questionnaires on sleep (ISI, MAPI, Functional Assessment of Chronic Illness Therapy—Fatigue [FACIT-Fatigue]). Follow-up information was collected until 30 September 2020.

The activities of OPAL Rheumatology Ltd have received overarching ethics approval from the University of NSW Human Research Ethics Committee (UNSW HREC), based on a patient opt-out arrangement (HC17799). This research protocol was approved by the UNSW HREC (HC180712).

### Patient-reported outcomes (PROs)

Validated PROs (ISI, MAPI, FACIT-Fatigue) were sent from the patients EMR within Audit4 and delivered to the patient’s email address at time intervals specified by their treating rheumatologist or completed in the clinic waiting room using a tablet or the patient’s smart phone. Completed questionnaires were encrypted and returned to the patient’s Audit4 EMR for review at the next consultation [12].

### Objectives and outcomes

The primary objective was to determine the prevalence of sleep disorders as measured by the number of patients with AS who were identified as being at risk of insomnia or obstructive sleep apnoea using the ISI or MAPI during the index period divided by the total number of patients with AS who had completed a survey.

The secondary objectives were to assess the relationship between demographic factors and sleep disturbance; to assess the impact of therapy for AS on sleep outcomes; to assess the prevalence of sleep disturbance in patients by AS therapy, specifically treatment with a tumour necrosis factor inhibitor (TNFi) or an interleukin 17A inhibitor (IL-17Ai), at index; and to assess the correlation between clinical signs and symptoms (measured by the BASDAI and FACIT-Fatigue), with sleep disturbance.

### Statistical methods

All patients with clinical data in the OPAL dataset that met the inclusion/exclusion criteria were included in the study. Assuming that 500 patients completed the questionnaires (of an approximate patient population with AS in the OPAL dataset of 3,800), then if the prevalence of sleep disturbance was 65% in either treatment group (based on previously published data [13]), the confidence interval around IL-17Ai users was calculated to be 51.2 to 78.8% and around TNFi users was calculated to be 60.5 to 69.5%. This assumes that IL-17Ai users made up 10% of the PRO completers, and TNFi users made up 90% of the PRO completers. The proportions of patients prescribed IL-17Ai vs TNFi was based analysis of prescribing patterns from the OPAL dataset.

Due to the potential biases in observational data, secondary analyses used a propensity score matching method to increase the comparability of the observed index characteristics of patients treated with IL-17Ai compared to TNFi. The propensity score is the conditional probability of receiving treatment (that is IL-17Ai vs TNFi) which was estimated using linear regression. We planned to use age group at index, sex, BASDAI score, duration of disease, concomitant therapy and line of therapy as covariates in the model. We planned to use a ratio of one patient prescribed IL-17Ai to every ten patients prescribed TNFi, which reflected the current treatment usage patterns at the time of study initiation. We assessed the success of matching by comparing the standardized differences in means and proportions between groups, with differences of more than 0.1 considered substantial differences [14]. We were unable to find suitable matches on all the planned covariates due to missing data or inadequate matching; therefore, the propensity score matching was conducted using sex, age category and disease duration. BASDAI category was missing for 183 (37%) of patients, and suitable matches on concomitant medication and line of therapy were not possible. We used a ratio of one user of IL-17Ai to every two users of TNFi. Further matching was not possible due to inadequate matches being available within the dataset.

Patients were defined as having no clinically significant insomnia (ISI score ≤ 7), subthreshold insomnia (ISI > 7 and ≤ 14), moderate severity clinical insomnia (ISI score > 14 and ≤ 21) or severe clinical insomnia (ISI score > 21) [15]. Patients were considered to have poor disease control if their BASDAI score was ≥ 4 [16]. The MAPI is a score for predicting sleep apnoea in patients [17]. Scores range from 0 to 1, with higher scores associated with an increased probability of sleep apnoea. MAPI values higher than 0.5 are suggestive of clinical apnoea [17].

The association of demographic factors on sleep disturbance (ISI category, MAPI) was assessed using ordinal or logistic regression (as appropriate). Covariates included in the model were age (category), sex, weight, body mass index (BMI), symptom duration, BASDAI score (< 4 vs ≥ 4) and use of steroids (no vs yes), non-steroidal anti-inflammatory drugs (NSAIDs; no vs yes) and bDMARDs (IL-17Ai vs TNFi).

All analyses were conducted in Stata MP V16.1 for Mac (StataCorp, Texas Station, USA). Missing data were not imputed. All tests were conducted two sided and values of *p* < 0.05 were considered statistically significant.

## Results

### Participants

Of the 5,323 patients with AS in the OPAL dataset, 629 (11.8%) were sent sleep questionnaires and 495 (9.3%) were included in the study (79% response rate), and the demographics are reported in Table 1. Of the 495 patients, 48 (9.7%) were receiving IL-17Ai, 395 (79.8%) were receiving TNFi and 52 (10.5%) were receiving other biological and non-biological treatments. For patients receiving TNFi, the majority were receiving adalimumab (*n* = 167/395, 42.3%) or etanercept (*n* = 92/395, 23.3%). A greater proportion of women were receiving IL-17Ai inhibitors compared to TNFi or other treatments (64.6%, 41.8% and 46.2%, respectively, *p* < 0.001), and less patients in the IL-17Ai group were receiving the drug as a first line biologic treatment compared to TNFi (64.6% vs 85.3%, *p* = 0.01). Median CRP was lower in the IL-17Ai group compared to other groups (2.0 in IL-17Ai compared to 3.0 in TNFi or 3.4 in other treatment, *p* = 0.026). There were no other clinically relevant differences in index demographics in the overall population.

**Table 1:** Patient demographics

Factor	Level	Value
*N*		495
Age at index (years), mean (SD)		48.3 (13.6) (*n* = 495)
Age category (years)	18–34 years	79 (16.0%)
	35–44 years	117 (23.6%)
	45–54 years	138 (27.9%)
	55–64 years	103 (20.8%)
	65–74 years	40 (8.1%)
	75–95 years	18 (3.6%)
Sex	Male	274 (55.4%)
	Female	220 (44.4%)
	Missing	1 (0.2%)
BMI category	Underweight	8 (1.6%)
	Normal weight	142 (28.7%)
	Overweight	144 (29.1%)
	Obese	168 (33.9%)
	Missing	33 (6.7%)
Symptom duration (months), mean (SD)		164.4 (151.4) (*n* = 495)
Line of bDMARD therapy	1	371 (74.9%)
	2	51 (10.3%)
	3	17 (3.4%)
	4	5 (1.0%)
	5	1 (0.2%)
	7	1 (0.2%)
	No prior bDMARD	49 (9.9%)
Duration of treatment (months), mean (SD)		57.9 (107.3) (*n* = 311)
BASDAI category	Optimal disease control (< 4)	256 (51.7%)
	Suboptimal disease control	56 (11.3%)
	Missing	183 (37.0%)
ESR, median (IQR)		6.0 (3.0, 14.0) (*n* = 399)
CRP, median (IQR)		2.8 (1.0, 4.2) (*n* = 409)
Depression	No	471 (95.2%)
	Yes	24 (4.8%)

*BASDAI*, Bath Ankylosing Spondylitis Disease Activity Index; *bDMARD*, biological disease modifying antirheumatic drug; *BMI*, body mass index; *IQR*, interquartile range; *SD*, standard deviation

In the propensity score matched set, 48 patients were prescribed IL-17Ai at index, and 94 were prescribed TNFi at index (Table 2). Patients in each group were well matched, with the exception of line of therapy, where there was a higher proportion of TNFi users in first line of bDMARD therapy compared to IL-17Ai users (82% vs 65%, *p* = 0.034).

**Table 2:** Patient demographics (propensity score matched population)

Factor	Level	TNFi	IL-17Ai	*p* value
*N*		94	48	
Age at index (years), mean (SD)		48.8 (12.8) (*n* = 94)	51.1 (14.7) (*n* = 48)	0.34
Age at index (years), median (IQR)		47.0 (40.0, 58.0) (*n* = 94)	48.0 (40.5, 61.5) (*n* = 48)	0.46
Age category (years)	18–34 years	11 (12%)	5 (10%)	0.47
	35–44 years	21 (22%)	12 (25%)	
	45–54 years	34 (36%)	13 (27%)	
	55–64 years	13 (14%)	9 (19%)	
	65–74 years	14 (15%)	6 (12%)	
	75–95 years	1 (1%)	3 (6%)	
Sex	Male	30 (32%)	16 (33%)	0.36
	Female	64 (68%)	31 (65%)	
	Missing	0 (0%)	1 (2%)	
BMI (kg/m^2), mean (SD)		30.7 (26.7) (*n* = 86)	31.0 (8.9) (*n* = 46)	0.94
BMI category	Underweight	2 (2%)	1 (2%)	0.094
	Normal weight	33 (35%)	8 (17%)	
	Overweight	24 (26%)	16 (33%)	
	Obese	27 (29%)	21 (44%)	
	Missing	8 (9%)	2 (4%)	
Symptom duration (months), mean (SD)		127.0 (116.5) (*n* = 94)	143.0 (149.8) (*n* = 48)	0.49
Symptom duration (months), median (IQR)		96.3 (35.9, 182.6) (*n* = 94)	75.7 (26.6, 249.6) (*n* = 48)	0.98
Line of bDMARD therapy	1	77 (82%)	31 (65%)	0.034
	2	8 (9%)	12 (25%)	
	3	7 (7%)	4 (8%)	
	4	2 (2%)	0 (0%)	
	5	0 (0%)	1 (2%)	
Duration of treatment (months), mean (SD)		61.0 (156.7) (*n* = 69)	23.7 (20.1) (*n* = 32)	0.18
BASDAI category	Optimal disease control (< 4)	43 (46%)	23 (48%)	0.89
	Suboptimal disease control	16 (17%)	8 (17%)	
	Missing	35 (37%)	17 (35%)	
ESR, mean (SD)		12.0 (13.5) (*n* = 81)	10.2 (8.8) (*n* = 41)	0.43
ESR, median (IQR)		8.0 (4.0, 15.0) (*n* = 81)	8.0 (4.0, 14.0) (*n* = 41)	0.68
CRP, mean (SD)		4.7 (7.3) (*n* = 81)	5.6 (6.9) (*n* = 41)	0.55
CRP, median (IQR)		2.5 (1.0, 4.2) (*n* = 81)	3.0 (2.0, 6.0) (*n* = 41)	0.15
Depression	No	90 (96%)	45 (94%)	0.60
	Yes	4 (4%)	3 (6%)	

*BASDAI*, Bath Ankylosing Spondylitis Disease Activity Index; *bDMARD*, biological disease modifying antirheumatic drug; *BMI*, body mass index; *IQR*, interquartile range; *SD*, standard deviation

### Proportion of patients with sleep disorders at index

At index, the mean ISI total score in the overall population was 8.6 ± 6.2. Moderate or severe clinical insomnia based on the ISI was present in 16% and 3.2% of patients, respectively. This was largely driven by reported difficulties staying asleep, dissatisfaction with sleep and sleep interference. The mean MAPI score at index was 0.4 ± 0.3, with 31.5% of evaluable patients (that is patients with a non-missing MAPI questionnaire) identified as being at risk of sleep apnoea. Few patients self-reported stopping breathing (rarely 10.9%, sometimes 5.1%, frequently 1.6% or always 1.6%). Moderate or severe clinical insomnia based on the ISI and a risk of sleep apnoea based on MAPI score > 0.5 was reported in 5.6% of patients. The mean FACIT-Fatigue total score at index was 36.1 ± 10.7. The greatest difficulties reported in FACIT-Fatigue subscales were with fatigue, tiredness, energy levels and interference with usual activities.

### Associations between therapy and sleep disturbance

In the overall population, there were statistically significant differences in the proportion of patients reporting the following symptoms on the ISI: difficulty staying asleep (severe or very severe problems: IL-17Ai 14.6%, TNFi 9.6%, other 26.9%, *p* = 0.002); satisfaction with their sleep (dissatisfied or very dissatisfied: IL-17Ai 20.8%, TNFi 23.5%, other 40.4%, *p* = 0.011); experiencing sleep interference (much or very much: IL-17Ai 18.7%, TNFi 14.7%, other 27.2%, *p* < 0.001); problems being noticeable to others (much or very much: IL-17Ai 12.5%, TNFi 8.8%, other 21.1%, *p* = 0.007); and worry or distress (much or very much: IL-17Ai 10.4%, TNFi 9.9%, other 12.4%, *p* = 0.012). However, there was no difference between the groups in the proportion of patients with clinically suspected moderate or severe insomnia (IL-17Ai 20.9%, TNFi 16.0%, other 28.9%, *p* = 0.079).

There were some treatment-related differences in the propensity score matched population: the mean MAPI index was higher in those with IL-17Ai at index (IL-17Ai 0.4 ± 0.3 and TNFi 0.3 ± 0.2, *p* = 0.046), and the proportion at risk of sleep apnoea based on the MAPI score was higher in those treated with IL-17Ai than those treated with TNFi (66% vs 54%, *p* = 0.051), although this did not reach statistical significance.

### Association between demographic factors on sleep disturbance

In the propensity score matched population, those with poor disease control (BASDAI ≥ 4) were seven times more likely (odds ratio [OR] 7.29, 95% CI 2.37 to 22.46, *p* = 0.001) to have a greater severity of insomnia symptoms as measured by ISI category than those with good disease control. Those taking steroids at index were almost four times more likely (OR 3.98, 95% CI 1.03 to 15.34, *p* = 0.045) to experience greater sleep disturbance than those not taking steroids. There were 87 patients included in this model. No other factors were significant (Table 3). It should be noted that model fit was relatively poor (McFadden’s pseudo *R*-squared 0.2516), the model is likely over specified (that is the number of covariates in the model is not supported by the number of patients included in it), and there were wide confidence intervals.

**Table 3:** Relationship between ISI category and demographic factors in the propensity score matched population, ordinal regression (*n* = 87)

Demographic factor	OR	95% CI	*p* value
Age			
18–34 years	Reference		
35–44 years	0.24	0.03–1.87	0.172
45–54 years	0.39	0.06–2.43	0.313
55–64 years	0.05	0.01–0.54	0.013
65–74 years	0.25	0.03–2.00	0.193
75–94 years	1.06 × 10^−7^		0.990
Sex			
Male	Reference		
Female	1.86	0.53–6.51	0.332
Other	(Omitted)		
BMI			
Underweight	Reference		
Normal weight	2.09	0.08–55.06	0.658
Overweight	11.59	0.93–341.2	0.156
Obese	13.37	0.47–377.85	0.128
Symptom duration (months)	1.00	0.97–1.00	0.891
BASDAI			
< 4	Reference		
≥ 4	7.29	2.37–22.46	0.001
Treatment			
TNFi	Reference		
IL-17Ai	1.33	0.48–3.66	0.582
Steroids used	3.98	1.03–15.34	0.045
NSAIDs used^a^	2.29	0.78–6.71	0.131
cDMARDs used^b^	0.99	0.30–3.34	0.992

^a^NSAIDs used if the patient was taking any one of celecoxib, piroxicam, naproxen, ibuprofen, indomethacin, diclofenac sodium or meloxicam at index. Comparison is to those not taking NSAID

^b^cDMARDs used if the patient was taking any one of methotrexate, leflunomide, azathioprine, sulfasalazine, hydroxychloroquine, 6-mercaptopurrine, cyclosporine, sodium aurothiomalate, olsalazine or balsalazide at index. Comparison is to those not taking cDMARD

*BASDAI*, Bath Ankylosing Spondylitis Disease Activity Index; *BMI*, body mass index; *cDMARD*, conventional disease modifying antirheumatic drug; *NSAID*, non-steroidal anti-inflammatory drug; *SD*, standard deviation

In the MAPI model, females were less likely (OR 0.00, 95% CI 0.00 to 0.13, *p* < 0.001) to be at risk of sleep apnoea; those reported as taking NSAIDs within the EMR were 12 times more likely to be at risk of sleep apnoea (OR 12.1, 95% CI 1.42 to 103.1, *p* = 0.022) compared to those not taking NSAIDs. No other factors were significant. Again, model fit was poor (McFadden’s pseudo *R*-squared 0.5518), and for some factors there are extremely wide confidence intervals, and the model is likely over specified (that is the number of covariates in the model is not supported by the number of patients within the model as it included only 83 patients).

### Correlation between clinical signs and symptoms with sleep disturbance

In patients administered TNFi at index, there were significant correlations between BASDAI and FACIT-Fatigue (*p* < 0.0001), BASDAI and ISI total score (*p* < 0.0001), and FACIT-Fatigue and ISI scores. In those treated with IL-17Ai, only the relationship between MAPI score and BASDAI scores (*p* = 0.04) and FACIT-Fatigue and ISI scores (*p* = 0.0001) were significant (Table 4).

**Table 4:** Pairwise correlations between MAPI, BASDAI, FACIT and ISI scores in the overall population

	IL-17Ai				TNFi				Other			
	MAPI category^a^	BASDAI category^b^	FACIT score^c^	ISI category^d^	MAPI category^a^	BASDAI category^b^	FACIT score^c^	ISI category^d^	MAPI category^a^	BASDAI category^b^	FACIT score^c^	ISI category^d^
MAPI score	1.0000				1.0000				1.0000			
BASDAI score	− 0.3985	1.0000			0.1080	1.0000			− 0.4082	1.0000		
	*p* = 0.0437				*p* = 0.0958				*p* = 0.2415			
FACIT score	0.1273	− 0.3152	1.0000		− 0.0203	− 0.4013	1.0000		− 0.3248	− 0.5436	1.0000	
	*p* = 0.4217	*p* = 0.1093			*p* = 0.7142	*p* < 0.0001			*p* = 0.0259	*p* = 0.1044		
ISI score	− 0.2882	0.2549	− 0.5711	1.0000	0.0244	0.3390	− 0.5927	1.0000	0.2845	0.5469	− 0.6245	1.0000
	*p* = 0.0642	*p* = 0.1664	*p* = 0.0001		*p* = 0.6549	*p* < 0.0001	*p* < 0.0001		*p* = 0.0500	*p* = 0.0658	*p* < 0.0001	

^a^MAPI score < 0.5 and ≥ 0.5; ^b^BASDAI score < 4 and ≥ 4; ^c^Total FACIT score (range 0 [worse fatigue] to 52); ^d^ISI category 0—no clinically significant insomnia, 1—subthreshold insomnia, 2—clinical insomnia (moderate severity), 3—clinical insomnia (severe)

*BASDAI*, Bath Ankylosing Spondylitis Disease Activity Index; *FACIT*, Functional Assessment of Chronic Illness Therapy; *ISI*, Insomnia Severity Index, *MAPI*, Multivariate Apnoea Prediction Index

In the both IL-17Ai and TNFi populations, MAPI score was significantly negatively correlated with response to BASDAI question 6 ‘*how long does your morning stiffness last from the time you wake up*’ (IL-17Ai − 0.45, *p* = 0.02; TNFi − 0.38, *p* < 0.0001). FACIT-Fatigue score was negatively correlated with BASDAI question 4 ‘*how would you describe the overall level of discomfort you have had from any areas tender to touch or pressure*’ (IL-17Ai: − 0.42, *p* = 0.03; TNFi: − 0.46, *p* < 0.0001). Insomnia symptoms as measured by ISI category were significantly associated with all BASDAI questions (Table 5).

**Table 5:** Pairwise correlation between BASDAI individual question and ISI category by treatment at index

		BASDAI q1	BASDAI q2	BASDAI q3	BASDAI q4	BASDAI q5	BASDAI q6
ISI category	IL-17Ai	0.3655	0.3846	0.3477	0.4194	0.4843	0.4439
		*p* = 0.0432	*p* = 0.0327	*p* = 0.0553	*p* = 0.0188	*p* = 0.0058	*p* = 0.0124
	TNFi	0.4078	0.3616	0.3986	0.3741	0.3499	0.2617
		*p* < 0.0001	*p* < 0.0001	*p* < 0.0001	*p* < 0.0001	*p* < 0.0001	*p* < 0.0001

ISI category 0—no clinically significant insomnia, 1—subthreshold insomnia, 2—clinical insomnia (moderate severity), 3—clinical insomnia (severe)

*BASDAI*, Bath Ankylosing Spondylitis Disease Activity Index; *ISI*, Insomnia Severity Index

## Discussion

### Overall findings

In this subset of patients with AS, 19.2% reported moderate to severe clinical insomnia, and 31.5% were identified as at risk of sleep apnoea. We found no significant differences in the incidence of insomnia or sleep apnoea by treatment type in our matched population (TNFi vs IL-17Ai), with the exception of the MAPI score which was significantly higher in those treated with IL-17Ai. Those with poor disease control were more likely to experience greater sleep disturbance than those with good disease control measured by BASDAI.

### Implications for research and practice

The proportions of patients who reported moderate to severe clinical insomnia or identified as at risk of sleep apnoea are within the range of previously reported estimates among AS patients [3, 4], but higher, particularly with respect to apnoea, than typical for healthy populations [18, 19]. The high prevalence of insomnia and sleep apnoea in this real-world population of patients with AS highlights the importance of actively assessing patients in routine clinical care for signs of sleep disorders and referring patients to sleep physicians for further assessment and treatment where indicated. Disturbed sleep, including multiple nocturnal awakenings, early morning awakening, sleep onset insomnia, difficulty awakening and daytime malfunctioning can leave patients with AS feeling fatigued [20]. These sleep disorders are noted to be more severe than those experienced by patients without AS [1]. While the present study was not designed to consider the impact of commencement of treatment for sleep disorders in people with AS, treatment of sleep disorders, such as sleep apnoea, has been associated with improvements in AS [21]. Treatment of the underlying AS can also improve sleep disturbance: for example, treatment with TNFi has been reported to improve sleep disturbance in patients with AS [22], although others report that improvements are only with sleep quality, and treatment with TNFi does not improve sleep measured via polysomnography [23]. This is possibly because the specific impact of AS on sleep depends on the underlying symptomology of AS. For example, pain is associated with poor sleep efficiency (the proportion of time attempting sleep that is actually spent asleep), while elevated CRP is associated with longer sleep latency [1]. TNFα and IL-17A have been reported to be important in the pathogenesis of obstructive sleep apnoea; it is possible that TNFα is more strongly associated with its development [24, 25].

We found that those with poor disease control were more likely to experience greater sleep disturbance than those with good disease control measured by BASDAI. This may reflect lesser control of the underlying inflammatory condition (for example levels of TNFα and IL-17A). There is a bidirectional relationship between sleep disturbance and disease control [20]: sleep disorders may contribute to emerging pain disorders, but pain may also lead to or exacerbate sleep disorders [26]. Unsurprisingly, we found that those patients taking concomitant steroids had increased levels of sleep disturbance. This is a well characterized association [27].

We also found that men with AS were at higher risk of sleep apnoea, which reflects the known sex epidemiology of sleep apnoea [28]. Others have reported that two to three times more men without AS are diagnosed with obstructive sleep apnoea compared to women [28]. We found no association between sleep disturbances and BMI, although this was likely confounded by sex, and the relationship between BMI and severity of obstructive sleep apnoea is likely complex [28].

### Strengths and potential limitations

Strengths of our study include that it is based on real-world clinical evidence collected at the point of care for patients with ankylosing spondylitis in Australia. The OPAL dataset is one of the largest datasets of patients with rheumatic diseases in the world [12]. In addition, OPAL captures multifaceted outcomes data from the patient perspective through a novel electronic patient-reported outcome (ePRO) delivery system which allows for health-related quality of life measures to be matched with clinical indices [12]. The patient-reported outcomes included in this study are clinically relevant for the initial assessment of sleep disturbance and sleep apnoea.

Our study has some potential limitations. This is a retrospective study based on data captured during routine clinical care. The analysis is therefore limited by the availability of data for secondary research in the dataset. The presence of missing data may also impact on the analysis; sample size, variables and study duration were selected to minimize the impact of missing data. Of the available patients with AS, only 9.3% were included in this study. Our assessment of sleep disorders was based on self-reported responses to sleep questionnaires and objective assessment via polysomnography, for example, was not conducted. It is possible therefore that the true estimate of sleep disorders may be lower or higher than that observed in our analysis. Additionally, the BASDAI is a subjective questionnaire, and further studies are needed to evaluate the association between sleep disturbance and objective signs of disease activity such as the ASDAS or c-reactive protein levels. Our regression modelling was hampered by poor fit, likely due to a combination of low sample sizes, and that sleep disturbance in this real-world population is likely multi-factorial and requires further investigation. For example, we did not assess the influence of exercise/physical activity on sleep as this information is not available in the dataset. Finally, we made no adjustments for multiple comparisons, and as such, it is possible that some of our statistically significant findings occurred by pure chance.

## Conclusions

In this real-world cohort of patients with AS, poor disease control was associated with sleep disturbance. Little difference in sleep disturbance was observed between TNFi and IL-17Ai treatment. Screening for insomnia, sleep apnoea and fatigue in routine clinical care may provide a more holistic view of the burden of this disease, and striving to reach low disease activity may have greater positive impacts on patient quality of life beyond traditional clinical disease activity measures.

## Data Availability

The OPAL dataset is held by OPAL Rheumatology. Data are available for research purposes upon application and approval by OPAL Rheumatology. All projects require HREC review.

## Code availability

Custom code in Stata MP v16.2.
